# Mapping the canine vector-borne disease risk in a Mediterranean area

**DOI:** 10.1186/s13071-020-04153-8

**Published:** 2020-06-03

**Authors:** Despoina Kostopoulou, Manuela Gizzarelli, Panagiota Ligda, Valentina Foglia Manzillo, Katerina Saratsi, Serena Montagnaro, Bettina Schunack, Annette Boegel, Matthias Pollmeier, Gaetano Oliva, Smaragda Sotiraki

**Affiliations:** 1 Veterinary Research Institute, Hellenic Agricultural Organization Demeter, 57001 Thermi, Thessaloniki, Greece; 2 Department of Veterinary Medicine and Animal Production, University of Naples Federico II, 80137 Naples, Italy; 3 Bayer Animal Health GmbH, Leverkusen, Germany

**Keywords:** *Anaplasma*, Dogs, *Ehrlichia*, Greek islands, *Leishmania*, Seroprevalence

## Abstract

**Background:**

The aim of this study was to determine exposure to vector-borne pathogens (VBPs) in populations of dogs living on Greek islands in the Ionian and Aegean seas.

**Methods:**

In total, 1154 dogs with different lifestyles and of varying ages and breeds were randomly sampled and examined for the presence of clinical signs compatible with canine vector-borne diseases (CVBDs). Blood was collected from each individual animal. For the detection of antibodies against *Leishmania* spp., the WITNESS® Leishmania test was performed, and positive samples were further examined with indirect enzymatic immunoassay (ELISA). Antibodies to *Borrelia burgdorferi*, *Ehrlichia canis* or *E. ewingii*, as well as *Anaplasma phagocytophilum* or *A. platys* were investigated using the Snap® 4Dx® Plus test. Positive *Ehrlichia* spp. and *Anaplasma* spp. samples were further examined using an indirect ELISA for further identification of the species.

**Results:**

In total, 25.6% of dogs were exposed to at least one of the pathogens investigated, with seroprevalences varying regionally. Of these seropositive dogs, 27.4% displayed clinical signs suggestive of CVBDs, such as cutaneous lesions, enlarged lymph nodes, pale mucous membranes, onychogryphosis and weight loss. The overall seroprevalence detected using the rapid tests was 15.3% for *Leishmania* spp., whereas 2.3% of the examined dogs were found to be positive for *Anaplasma* spp. and 7.5% for *Ehrlichia* spp. while *B. burgdorferi* was not detected. Twenty-four samples positive to *A. phagocytophilum* by ELISA were analysed by PCR for the presence of *Anaplasma* DNA. PCR and sequencing results showed the presence of *A. platys* DNA in 4 samples and *E. canis* DNA in 4 samples. The remaining samples (66.7%) were negative.

**Conclusions:**

In the present study, exposure of dogs to VBPs was shown in the geographical areas investigated. Results confirm that on Greek islands VBPs represent a constant health risk for both native and visiting dogs, suggesting the presence of distinct “hot-spots” of VBP infections on different islands. In order to reduce the risk of transmission and the spread to non-endemic regions, the protection of dogs through use of repellents and vaccines, together with owner education, seem to be of paramount importance.
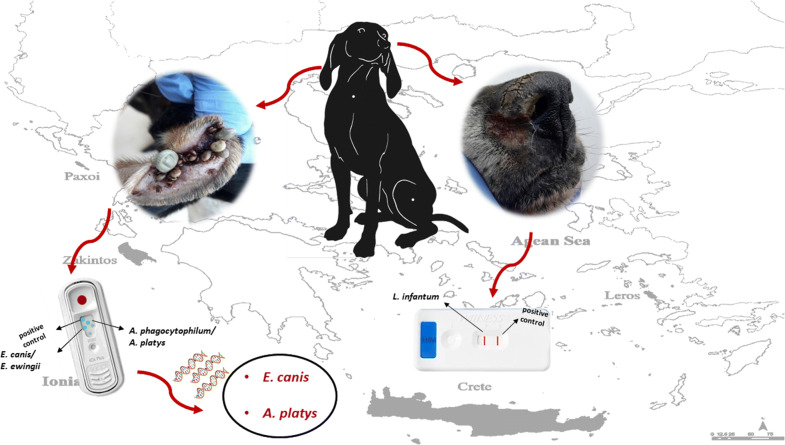

## Background

Vector-borne diseases (VBDs) represent a growing global threat, both in human and veterinary medicine, because of their constant spread from traditional geographical and temporal restraints to new areas, exposing new populations to previously unknown infectious agents and posing unprecedented challenges to veterinarians. The constantly changing epidemiology of VBDs is being influenced by different factors, the most important of which are climatic changes (affecting in many ways both vector arthropods and pathogen development rates) and increasing mobility of owners and their companion pet populations, supporting in fact pathogen and vector spread [1–4].

Canine vector-borne diseases (CVBDs) are caused by a range of pathogens transmitted to dogs by blood-feeding arthropods, e.g. ticks, fleas, mosquitoes and sand flies. The CVBDs commonly diagnosed are anaplasmosis, babesiosis, bartonellosis, borreliosis, dirofilariosis, ehrlichiosis and leishmaniosis. These diseases are characterized by a complex pathogenesis with a potentially fatal clinical course for the majority of cases with new pathogenic findings being uncovered every year [5]. In addition, several have a zoonotic potential with possible transmission to the human population [6–8]. It is important to recognize that the transmitted pathogens may also frequently originate subclinical infections that nevertheless render the host as a carrier and even a reservoir [9].

In view of the above considerations and in the context of human and animal health protection, there is an immense need to map the presence of canine vector-borne pathogens (CVBPs) in areas such as popular tourist destinations.

The Greek islands are amongst the most visited touristic places around the world, attracting millions of people per year (i.e. 19.8 million international airport arrivals from January to October 2018, with 4.5 million arrivals on Crete alone [10]), many of whom travel with their pets. Overall, located in the Mediterranean basin with a suitable environment for pathogen transmission and poorly managed stray dog populations, Greece has been repeatedly reported as an area highly endemic for various CVBDs [11–17]. However, landscape and microclimate conditions are not identical throughout the country and this stresses the need for comprehensive knowledge on the distribution and abundance of various CVBPs within native dog populations and across different islands, which currently is missing.

The aim of this study was to gain information on (i) the health risk for both native and visiting dogs, and (ii) the risk of pathogen distribution to new areas (i.e. *via* dogs travelling back from holidays or stray dogs being adopted and moved to various other countries in Europe), by determining the exposure of dog populations living on Greek islands in different geographical locations to CVBPs.

## Methods

### Study design

**Fig. 1:**
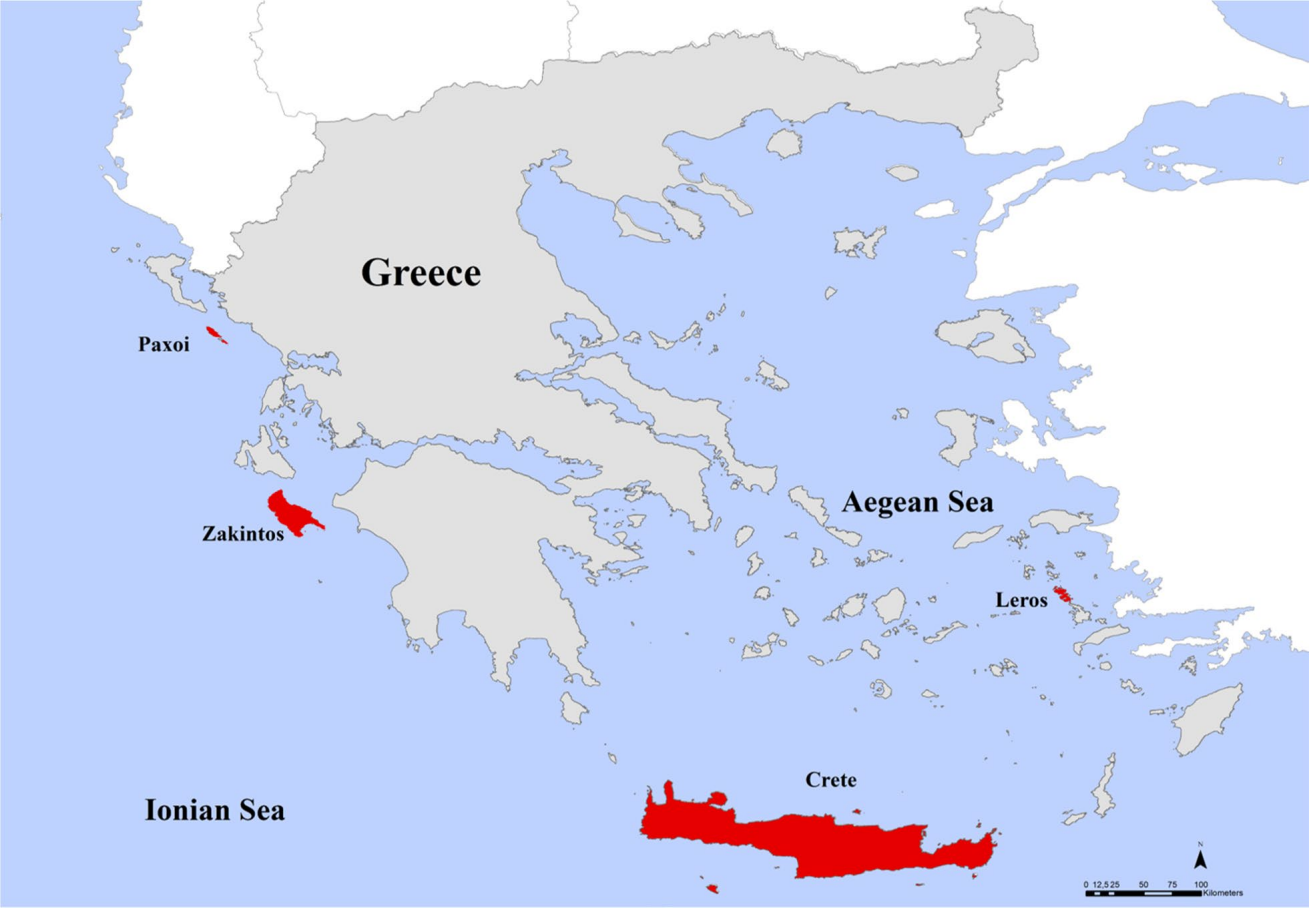
Map of Greece (the islands included in the study are marked in red)

A cross-sectional study was conducted in canine populations from different Greek Islands in order to evaluate the seroprevalence of CVBPs. In total, 4 islands were selected based on their geographical location [(i) situated in both Ionian and Aegean seas; (ii) covering areas West to East of the country; (iii) having different landscapes and climatic conditions], the size of their native dog population (traditionally islands like Leros and Paxoi despite their size are known for the high hunting dog population), and previous records (published or personal communication) of CVBD presence [11, 12, 14–17]. Altogether, 1154 dogs with different lifestyles (indoors/outdoors), irrespective of age and breed were randomly sampled and examined for the presence of clinical signs suggestive of CVBDs. Regarding the specific locations, 690 dogs were enrolled from Crete, 270 from Leros (located in the South Aegean Sea), 124 from Paxoi and 70 from Zakinthos (both located in the Ionian Sea) (Fig. 1). In Crete, due to its size (both in total surface and human/dog population) 3 sampling areas were included, representing 3 of the 4 Cretan counties (i.e. from the west to the east: Chania, Rethymno and Heraklion).

A blood sample of approximately 5 ml was collected from each individual animal equally divided into a gel and clot activator, and an EDTA tube. The samples were immediately maintained at 4 °C. All samples were examined within 2 days of collection.

### Serology

For the detection of antibodies against *Leishmania infantum*, the WITNESS® Leishmania test (Rapid Immuno Migration (RIM™) test; Zoetis Diagnostics, Parsippany, USA) was performed. Positive samples were further examined with an indirect enzymatic immunoassay (ELISA) for the detection of specific antibodies to *L. infantum* (INGEZIM® Leishmania; Ingenasa, Madrid, Spain).

Antibodies against *Borrelia burgdorferi*, *Ehrlichia canis/E. ewingii*, as well as *Anaplasma phagocytophilum/A. platys* were investigated with the Snap® 4Dx® Plus test (IDEXX Laboratories, Westbrook, USA). Samples which tested positive for *Ehrlichia* spp. and *Anaplasma* spp. with the rapid test were further examined using an indirect ELISA for the detection of antibodies specific to *E. canis* (INGEZIM® Ehrlichia; Ingenasa) and *A. phagocytophilum* (Anaplasma-ELISA Dog; AFOSA GmbH, Blankenfelde-Mahlow, Germany) respectively. All the procedures above were performed following the manufacturers’ instructions.

### Molecular analyses

Samples found positive for the presence of *Anaplasma* antibodies using ELISA, were further investigated with molecular techniques to identify the species of *Anaplasma*.

Genomic DNA was extracted from positive EDTA-blood samples using the DNeasy Blood & Tissue Kit (Qiagen GmbH, Hilden, Germany), according to the manufacturer’s instructions.

PCR was performed to amplify a 345 bp fragment of the *16S* rRNA gene [18] of various species including *E. canis*, *E. chaffeensis*, *E. muris*, *E. ruminantium*, *A. phagocytophilum*, *A. platys*, *Anaplasma marginale*, *Anaplasma centrale*, *Wolbachia pipentis*, *Neorickettsia sennetsu*, *Neorickettsia risticii* and *Neorickettsia helminthoeca*, according to previously described thermal-cycling conditions [19]. A positive (*A. phagocytophilum* reference DNA sample) and a negative (PCR grade water) control were included in each PCR run. Amplification products were visualized on 1.5% agarose gels stained with ethidium bromide. PCR products were sent to a commercial service (CeMIA SA, Larissa, Greece) for purification and sequencing on both strands (Sanger sequencing). The results were assembled with Seqman 8.1 software (Lasergene DNASTAR, Madison, WI, USA). Assembled sequences were aligned using the Basic Local Alignment Tool (BLAST) and compared with reference sequences using the MegAlign (Lasergene DNASTAR).

### Statistical analysis

As age was deemed an important variable, dogs included were split into age groups (≤ 3; > 3–6; > 6–12; > 12 years-old). In addition, lifestyle (indoor or outdoor) and location of the animal were used as variables to calculate proportions for each of the pathogen species identified and summarized descriptively.

Seroprevalence was calculated based on the rapid test results, which were also used for further analyses.

Analyses of the binary variables (negative or positive) were performed using a generalized linear mixed model (GLIMMIX) and a logit link [20]. The statistical model included lifestyle, location, age category, the interaction of lifestyle and location as fixed effects as well as the 2-way interactions of lifestyle * age category, and location * age category, and the 3-way interaction of lifestyle * location * age category as random effects. If any of the fixed factors were found to be significant (*alpha* = 0.05 for all analyses), then pairwise comparisons of the possible combinations were performed at the 0.05 level. SAS® statistical software version 9.4 was used for all analyses.

## Results

The majority of dogs sampled were 3 years-old or younger (45.7%) and the sample size included a comparable number of dogs living indoors or outdoors (Tables 1, 2 and 3).

**Table 1:** *Leishmania* spp. seroprevalence (rapid test results) analyzed by age, lifestyle and location

Variable	Descriptive statistics			Model-adjusted statistics^a^		
	*n*	Positive (%)	95% CI (%)	Mean ± SE (%)	95% CI (%)	*P*-value
Total	1154	176 (15.3)	13.2–17.5			
Age (years)						
≤ 3	527	69 (13.1)	10.3–16.3	13.3 ± 2.7	6.8–24.2	0.2793
> 3–6	316	49 (15.5)	11.7–20.0	16.2 ± 3.4	8.1–29.9	
> 6–12	292	52 (17.8)	13.6–22.7	20.3 ± 4.0	10.4–36.0	
> 12	19	6 (31.6)	12.6–56.6	33.7 ± 12.7	7.7–75.6	
Lifestyle						
Indoors	550	65 (11.8)	9.2–14.8	18.1 ± 3.1	10.2–30.1	0.5233
Outdoors	604	111 (18.4)	15.4–21.7	21.9 ± 5.4	9.3–43.4	
Location						
Crete*	690	95 (13.8)	11.3–16.6	14.9 ± 2.7	9.7–22.2	0.0051*
Leros*	270	20 (7.4)	4.6–11.2	9.3 ± 2.6	4.7–17.4	
Paxoi*	124	41 (33.1)	24.9–42.1	38.4 ± 6.2	25.5–53.3	
Zakinthos	70	20 (28.6)	18.4–40.6	25.5 ± 11.1	8.2–56.8	

^a^Model-adjusted for random effects and sample size

*Significant difference (*P* < 0.05) between Paxoi and Crete/Leros islands

*Abbreviations*: LSM, least square means; SE, standard error; CI, confidence interval

**Table 2:** *Ehrlichia canis*/*E. ewingii* seroprevalence (rapid test results) analyzed by age, lifestyle and location

Variable	Descriptive statistics			Model-adjusted statistics^a^		
	*n*	Positive (%)	95% CI (%)	LSM ± SE (%)	95% CI (%)	*P*-value
Total	1154	87 (7.5)	6.1–9.2			
Age (years)						
≤ 3	527	36 (6.8)	4.8–9.3	6.8 ± 1.1	5.0–9.3	0.4245
> 3–6	316	28 (8.9)	6.0–12.6	8.9 ± 1.6	6.2–12.5	
> 6–12	292	20 (6.8)	4.2–10.4	6.8 ± 1.5	4.5–10.4	
> 12	19	3 (15.8)	3.4–39.6	15.8 ± 8.4	5.2–39.2	
Lifestyle						
Indoors	550	45 (8.2)	6.0–10.8	8.2 ± 1.2	6.2–10.8	0.9994
Outdoors	604	42 (7.0)	5.1–9.3	7.0 ± 1.0	5.2–9.3	
Location						
Crete	690	47 (6.8)	5.0–9.0	7.6 ± 1.6	5.0–11.5	0.0014*
Leros*	270	40 (14.8)	10.8–19.6	18.2 ± 3.6	12.2–26.3	
Paxoi	124	0 (0)	0–2.9	0.0 ± 0.0	0–2.9	
Zakinthos	70	0 (0)	0–5.1	0.0 ± 0.0	0–5.1	

^a^Model-adjusted for random effects and sample size

*Significant difference (*P* < 0.05) between Leros and the other islands

*Abbreviations*: LSM, least square means; SE, standard error; CI, confidence interval

**Table 3:** *Anaplasma* spp. seroprevalence (rapid test results) analyzed by age, lifestyle and location

Variable	Descriptive statistics			Model-adjusted statistics^a^		
	*n*	Positive (%)	95% CI (%)	LSM ± SE (%)	95% CI (%)	*P*-value
Total	1154	26 (2.3)	1.5–3.3			
Age (years)						
≤ 3	527	7 (1.3)	0.5–2.7	1.3 ± 0.5	0.6–2.8	0.2321
> 3–6	316	8 (2.5)	1.1–4.9	2.5 ± 0.9	1.3–5.0	
> 6–12	292	10 (3.4)	1.7–6.2	3.4 ± 1.1	1.9–6.3	
> 12	19	1 (5.3)	0.1–26.0	5.3 ± 5.1	0.7–29.4	
Lifestyle						
Indoors	550	13 (2.4)	1.3–4.0	2.4 ± 0.6	1.4–4.0	0.9997
Outdoors	604	13 (2.2)	1.2–3.7	2.2 ± 0.6	1.3–3.7	
Location						
Crete	690	13 (1.9)	1.0–3.2	1.9 ± 0.5	1.1–3.2	0.2969
Leros	270	11 (4.1)	2.1–7.2	4.1 ± 1.2	2.3–7.2	
Paxoi	124	1 (0.8)	0–4.4	0.8 ± 0.8	0.1–5.5	
Zakinthos	70	1 (1.4)	0–7.7	1.4 ± 1.4	0.2–9.5	

^a^Model-adjusted for random effects and sample size

*Abbreviations*: LSM, least square means; SE, standard error; CI, confidence interval

During the initial screening by rapid test, the seropositivity of *L. infantum*, *E. canis/E. ewingii* and *A. phagocytophilum/A. platys* was recorded whereas all samples were found seronegative for *B. burgdorferi.* In total, 25.6% of the animals were seropositive for at least one pathogen and 2.2% of the animals co-seropositive for 2 or more pathogens. Of the seropositive dogs, 27.4% displayed clinical signs suggestive of CVBDs, such as cutaneous lesions, enlarged lymph nodes, pale mucous membranes, onychogryphosis and weight loss.

Specifically, for the dogs tested in the different counties of Crete, the percentages of samples found positive in the different tests were for: (i) Chania: 11.4% and 7.4% in the rapid test and the ELISA, respectively, for *L. infantum*; 0% in both tests for *E. canis/E. ewingii* and 0.5% in both tests for *A. phagocytophilum/A. platys*; (ii) Rethymno: 6.2% in both tests for *L. infantum*; 0% in both tests for *E. canis/E. ewingii*; and 0.5% in both tests for *A. phagocytophilum/A. platys*; and (iii) Heraklion: 16.4% and 8.5% according in the rapid test and the ELISA, respectively, for *L. infantum*; 11.4% in both tests for *E. canis/E. ewingii*; and 33.5% in both tests for *A. phagocytophilum/A. platys.* Crete was considered as one location for the needs of further analyses.

Specific results per pathogen are presented below.

### *Leishmania* spp.

A total of 176 out of 1154 serum samples tested positive for antibodies against *L. infantum* with the rapid test (15.3%, 95% confidence interval (CI): 13.2–17.3%). No statistically significant association was found in relation to seropositivity and lifestyle or age of animals. Location was the only factor found to be statistically important. With the rapid test a statistically significantly different number of dogs tested positive on Paxoi (model adjusted prevalence of 38%) compared to Crete and Leros, while the number of positive dogs from Zakinthos did not differ statistically from any of the other islands (Table 1).

Using the ELISA to confirm the 15.3% samples positive in the rapid test resulted in a reduced overall seroprevalence for *L. infantum* of 9.2%. This means overall 61.7% of the rapid test results were confirmed positive with the ELISA, in detail 66.7% for Zakinthos, 58.8% for Crete, 49.8% for Paxoi and 94% for Leros.

### *Ehrlichia* spp.

A total of 87 out of 1154 serum samples tested positive for *Ehrlichia* antibodies with the rapid test (7.5%, 95% CI: 6.1–9.2%). No statistically significant association was found in relation to lifestyle and age, even though prevalence increased with age. Location was the only statistically significant factor. On the island of Leros a significantly greater number of dogs were found positive (model adjusted prevalence of 18%) than on the other islands (Table 2). All samples positive in the rapid tests for *Ehrlichia* spp. were found to be seropositive for *E. canis* by ELISA.

### *Anaplasma* spp.

Twenty-six out of 1154 dogs were positive for *Anaplasma* spp. in the rapid test (2.3%, 95% CI: 1.5–3.3%). There were no statistically significant differences in relation to lifestyle, age and location of the animals (Table 3). The highest seroprevalence of *Anaplasma* positive dogs was observed on the island of Crete. All but 2 samples positive in the rapid tests for *Anaplasma* spp., were found to be seropositive for *A. phagocytophilum* by ELISA. In order to further investigate the presence of *A. phagocytophilum*, all 24 samples positive by ELISA (1 from Paxoi, 1 from Zakinthos, 11 from Leros and 11 from Crete) were analysed by PCR for the presence of *Anaplasma* DNA. None of the samples tested showed the presence of *A. phagocytophilum* DNA. However, PCR and sequencing showed the presence of *A. platys* DNA in 4 samples (2 from Leros and 2 from Crete), and *E. canis* DNA in 4 samples (3 from Leros and 1 from Crete). The remaining samples (66.7%) were negative for any of the above pathogens. Representative sequences obtained for *A. platys* and *E. canis* were deposited in the GenBank database under the accession numbers MN922608-MN922611.

## Discussion

In the present study, exposure of dogs to vector-borne pathogens was shown in all geographical areas investigated with the prevalence for different pathogens varying regionally.

### Leishmania infantum

*Leishmania infantum* was endemic on all islands, being the most prevalent agent with an overall prevalence of 15.3% based on the rapid test and 9.2% after specification with ELISA. The prevalence was lower than results found in earlier nation-wide studies, which showed a mean prevalence as high as 22.09% [11, 12]. In these previous studies, the seroprevalence of *L. infantum* found in dogs in Corfu, an island in the Ionian Sea close to Paxoi, was 50.2%, whereas in our sample from Paxoi it was 33.9% by the rapid test and 16.9% by ELISA. Accordingly, the prevalence previously reported for islands located in the South Aegean Sea ranged from 10.7% to 42% [11, 12, 17], rates which were to some extent higher than recorded in the present study (30% and 20% for Crete and 6.7% and 6.3% for Leros in the rapid test and ELISA, respectively).

One reason for such differences might be the diverse screening approaches used. In the present study, screening was referring to seroprevalence which was at first defined by the rapid test followed by an ELISA (cut-off titre 1:100) for positive results. In contrast, seroprevalence using an IFAT test (cut-off titre ranging from 1:40 to 1:160) and a questionnaire approach (based on confirmed laboratory of clinically cases of leishmaniosis) was used in previous studies [11, 12]. However, the rates observed here could also be attributed to a real reduction in the abundance of *Leishmania*, due to better awareness to the disease and better compliance to preventive measures by dog owners. It is worth mentioning that, in the meantime, the first relevant vaccine (CaniLeish®; Virbac, Carros, France, authorized in the European Union in 2011), and an increasing number of sand fly repellents have been introduced to the market.

Regarding the geographical differences, it seems that the exposure of dogs to *L. infantum* is higher on the Ionian Sea islands, especially on Paxoi. This is in agreement with previous observations regarding the geographical location of *Leishmania-*infected dogs. Within Crete, the highest seroprevalence was recorded in Heraklion county as also previously reported [11, 12].

Previous records on the sand fly fauna in Greece have already demonstrated a rich population of sand fly species on all of the studied islands, which includes important vectors of *Leishmania* spp. [11, 21]. However, islands in the Ionian Sea could represent a more favourable environment for both sand flies and *Leishmania* due to the different climatic conditions. Regardless of both areas covering a similar latitudinal range and having a typical Mediterranean climate, precipitation levels are higher on the Ionian islands (with mean precipitation of approximately 1000 mm annually) compared to the Aegean islands (with mean precipitation of approximately 600 mm annually), according to World Clim-Global climate data [22]. As previously documented by Ntais et al. [11] animals living in areas with higher annual rainfall, and low and medium values of mean wind speed had a higher risk of exposure to *Leishmania*. The high wind speed, influencing sand fly activity, could also be a reason for the lower seroprevalence found in Leros [23].

In general, quantitative ELISA-based tests perform better than qualitative immunochromatographic rapid tests. While both tests have a proven high specificity, the sensitivity of the rapid test used in the present study (WITNESS® Leishmania, Zoetis Diagnostics) is significantly lower (i.e. 0.58 *versus* 0.76) compared to the ELISA used (INGEZIM® Leishmania) [24]. Therefore, a lower number of seropositive dogs were detected when positive rapid test results were confirmed with the quantitative ELISA, resulting in 50% lower infection rates in Paxoi. This could also be due to antibody titres being below the ELISA cut-off (the standard cut-off value for quantitative ELISA is set at 1:100).

It is important to mention that although the diagnostic tests used in this study were aiming to detect specific antibodies against *L. infantum*, they are not always distinctive between antibodies against this species and *L. major* and/or *L. tropica* [25]. However, during clinical examinations, no clinical signs similar to those reported for *L. major*/*L. tropica* disease in dogs were recorded [26]. Due to previous records of *L. tropica* (and the competent sand fly vector) in Greece and its capacity to infect dogs, this point should be further investigated, especially since *L. major*/*L. tropica* cause anthroponotic cutaneous leishmaniasis [11, 27].

### *Ehrlichia* spp.

*Ehrlichia* spp. were diagnosed only in dogs living more to the East (i.e. Crete and Leros), with the prevalence being significantly higher in Leros. Even within the island of Crete there was an obvious tendency of a higher prevalence to the East (Heraklion showed highest prevalence for *Ehrlichia* and *Anaplasma*). Moreover, *Ehrlichia* seropositive dogs were not identified on the Ionian Sea islands. In the present study, the overall seroprevalence recorded was lower than that found in studies recently conducted in the whole of Greece (a prevalence of approximately 12.5%) with similar sample size and methods applied [14, 15]. In these studies, however, the majority, if not all, of samples originated from mainland Greece. In another recent study with a remarkable number of dogs (*n *= 200), a similar to the present data from Aegean islands *Ehrlichia* spp. seropositivity was found (9.5%) [16]. The above data further support the observation in this study that the exposure of dogs to *Ehrlichia* spp. varies significantly within the country, a fact which could be attributed to microhabitat conditions favouring (or not) both the vector and the pathogen. It is quite interesting to note that a recent study testing ixodid ticks (i.e. *Rhipicephalus sanguineus* (*sensu lato*), *R. turanicus*, *Haemaphysalis parva*, and *H. concinna*) from mainland Greece for the presence of VBP could not confirm the presence of *Ehrlichia* DNA [13].

### *Anaplasma* spp.

*Anaplasma* spp. were detected at all collection sites. Using the rapid test (SNAP® 4Dx® plus, IDEXX Laboratories), which detects both *A. phagocytophilum* and *A. platys*, a high number of samples were identified as *A. phagocytophilum/A. platys*, and later confirmed to be *A. phagocytophilum* by a specific quantitative ELISA. This high positivity for *A. phagocytophilum*, especially in Crete, was surprising insofar as the predominant tick species in Greece is *R. sanguineus* (*s*.*l*.), which is the vector for *A. platys* [28]. While *A. phagocytophilum* has been previously reported from *Rhipicephalus bursa* ticks in Greece [29], as well as from *Rhipicephalus* spp. ticks from other areas, indicating that there is a potential also for these ticks to play a role in the transmission of *A. phagocytophilum* [30]. The primary tick vector for *A. phagocytophilum*, *Ixodes ricinus*, is only rarely found in Greece. *Ixodes ricinus* ticks are primarily associated with shrubs and forests with deciduous trees; therefore, they can be found around the forested zones in the north, but the hot and dry semi-arid environment around studied areas does not represent the optimum conditions for this species.

Cross-reactivity between *A. phagocytophilum* and *A. platys* in serological tests has previously been shown by testing seropositive *A. phagocytophilum* samples with other, more sensitive techniques like PCR [31]. Therefore, in order to further investigate the presence of *A. phagocytophilum* within the study population, samples which tested positive for *A. phagocytophilum* by ELISA were further analysed by PCR. As a result, *A. phagocytophilum* DNA was not detected at any of the samples tested positive by ELISA. Instead, the presence of *A. platys* was confirmed in four of these samples and also the presence of *E. canis* DNA was detected in four other samples, which however, were also found to be positive for antibodies against *E. canis* by the species-specific ELISA. The failure to detect *Anaplasma* DNA by PCR could be due to chronic infections, in which parasites are not always present in the blood, or to cross-reactions leading to false positive results in serological testing [31]. Similar to the results for *Ehrlichia*, the overall seroprevalence recorded in the present study was lower than that in a recent study where samples originated mainly from mainland Greece [14]. Likewise, in the study including samples from Aegean islands, *Anaplasma* spp. were recorded in only 1% (2 out of 200 samples); however, *A. phagocytophilum* was isolated in one of the samples by PCR [16]. The presence of *Anaplasma* spp. DNA was confirmed in ticks collected from dogs from various locations in Greece and identified at the species level [32] as either *A. platys* [13] or *A. phagocytophilum* and *A. platys* [29].

### *Borrelia* spp.

None of the dogs examined were seropositive for *Borrelia* spp., which is in agreement with the very low prevalences recorded in whole of Greece [14, 15]. Within a given area, the risk of animal/human infection with *B. burgdorferi* (*s.l*.) is determined by the local abundance and infection of vector ticks and the wild vertebrate hosts. Therefore, this result was expected since *B. burgdorferi* (*s.l*.) is transmitted by ticks of the *I. ricinus* complex. *Ixodes ricinus* is only rarely found in Greece, and recent surveys also failed to identify the presence of *Borrelia* in ticks in Greece [13, 28, 29, 32, 33].

## Conclusions

This study confirms that dogs living on different Greek islands are largely exposed to CVBP. With different “hot-spots” of higher abundance of a specific pathogen in certain regions, more detailed knowledge on what drives the development of such “hot-spots” is necessary. Unfortunately, at this current time there is neither clear and definitive information on the ectoparasite species abundance in the different locations, nor on the use of preventatives against ticks or sand flies which would contribute to the understanding of these findings. The seroprevalence of CVBPs reported here represent a constant health risk for both native and visiting dogs, and in some cases may amplify the risk for human infection. Additionally, a major threat is posed by the potential spread of both vectors and pathogens, endemic in southern Europe to northern, non-endemic regions of Europe and beyond. However, in order to define this threat, the infection pressure (i.e. as calculated by the seroprevalence of native dogs) and time of exposure to it (e.g. days in the specific environment), in order for naïve dogs to become infected, need to be better understood, since there are studies that claim the risk for infection is low especially during a limited single stay in endemic countries [3]. In order to reduce the risk of transmission and the spread to non-endemic regions, the protection of dogs together with owner education, are of paramount importance.

## Data Availability

All data generated or analysed during this study are included in this published article. The raw datasets used during the present study are kept in the Veterinary Research Institute (VRI) of the Hellenic Agricultural Organization - DEMETER and are available upon reasonable request. Representative sequences for *A. platys* and *E. canis* were deposited in the GenBank database under the accession numbers MN922608-MN922611.

## Abbreviations

BLAST: basic local alignment tool
CI: confidence interval
CVBD: canine vector-borne disease
CVBP: canine vector-borne pathogen
EDTA: ethylenediamine tetra acetic acid
ELISA: enzyme-linked immunosorbent assay
IFAT: indirect fluorescent antibody test
PCR: polymerase chain reaction
rRNA: ribosomal RNA
SE: standard error
VBD: vector-borne disease
VRI: Veterinary Research Institute
